# Management of depression utilizing Traditional Chinese Medicine

**DOI:** 10.3389/fphar.2026.1763214

**Published:** 2026-04-20

**Authors:** Rong Ma, Junrui Ma, Hushan Zhang

**Affiliations:** 1 Zhaotong Health Vocational College, Zhaotong, China; 2 Yunnan University of Traditional Chinese Medicine, Kunming, China; 3 The First Affiliated Hospital of Yunnan University of Traditional Chinese Medicine, Kunming, China; 4 Anning People’s Hospital Affiliated to Kunming University of Science and Technology, Kunming, China

**Keywords:** depression, medicine, research status and progress, Traditional Chinese Medicine (TCM), treatment

## Abstract

Depression is a major global health concern, characterized by profound mental and physical debilitation that severely impairs quality of life. The World Health Organization projects that it will become the second-leading cause of global disease burden by 2030, underscoring the urgent need for effective therapeutic strategies. However, the pathogenesis of depression remains incompletely elucidated, and first-line pharmacological treatments, such as selective serotonin reuptake inhibitors (SSRIs), are frequently limited by delayed clinical onset, suboptimal response rates, and notable adverse effects. In contrast, traditional Chinese medicine (TCM), with its holistic philosophy and potential for multi-target modulation, offers a promising complementary approach. This review systematically synthesizes contemporary research on TCM for depression, covering botanical drug formulas, single medicinal botanical drugs, and their purified bioactive metabolites. We critically evaluate the proposed mechanisms—spanning monoaminergic regulation, modulation of neurotrophic factors, anti-inflammatory activity, and interactions between the microbiota-gut-brain axis—and discuss the associated translational challenges and future research directions.

## 1 Introduction

### 1.1 Depression

Depression constitutes a major cause of global disability, manifesting through core symptoms such as persistent low mood and anhedonia, alongside a range of other clinical features including fatigue, changes in appetite or weight, and sleep disturbances (Anderson et al., 2024). Additional common manifestations include cognitive deficits (e.g., diminished concentration) and psychological symptoms such as excessive guilt or feelings of worthlessness. Without effective intervention, depression can progress to its severe form, Major Depressive Disorder (MDD), which is associated with a markedly elevated risk of self-harm and suicide (Monroe and Harkness, 2022; Marwaha et al., 2023; Zhou et al., 2024).

Data from the World Health Organization (WHO) reveals that nearly one billion individuals across the globe are affected by mental disorders, with depression impacting over 350 million of these cases. During the COVID-19 pandemic, there was a rapid increase in the rates of depression and anxiety worldwide (COVID-19 Mental Disorders Collaborators, 2021). Concurrently, the “Blue Book on National Depression for 2023” discloses that in China alone, 95 million people suffer from depression, with approximately 280,000 individuals committing suicide each year. These alarming statistics highlight an urgent societal need to address this critical issue.

## 2 Objective of the study

First-line pharmacological treatments for depression, such as selective serotonin reuptake inhibitors (SSRIs), are effective in many patients but have significant limitations: delayed onset, variable response rates, and a range of adverse effects that may compromise treatment adherence (Chen et al., 2019). This unmet medical need has driven the exploration of complementary therapeutic options.

Traditional Chinese medicine (TCM) provides a unique diagnostic and therapeutic paradigm centered on holistic assessment and multi-target interventions. A growing body of preclinical and clinical evidence indicates that certain TCM therapies exert antidepressant effects and may have a distinct tolerability profile compared with conventional Western medicines (Serretti et al., 2004; Sarko, 2000; Li et al., 2019). Therefore, a systematic and critical evaluation of the available scientific evidence supporting TCM for depression is warranted.

This review aims to summarize the mechanisms of action, efficacy, and limitations of representative TCM botanical drugs, herbal formulas, and purified metabolites, and to assess their potential role in the modern management of depression.

## 3 Materials and methods

We conducted this review in accordance with the principles of a systematic narrative review. We designed the search and selection process to comprehensively identify and critically evaluate the literature on the antidepressant effects of Traditional Chinese Medicine (TCM), while giving due consideration to ethnopharmacological best practices.

### 3.1 Search strategy

A systematic literature search was performed across multiple electronic databases from their inception until December 2025. Databases included PubMed, Web of Science, EMBASE, Cochrane Library (international), as well as China National Knowledge Infrastructure (CNKI), Wanfang Data, and VIP Database (Chinese). The search strategy utilized a combination of Medical Subject Headings (MeSH) terms and free-text keywords. The core search concepts included: (1) Depression (“depression,” “major depressive disorder”); (2) Intervention (“traditional Chinese medicine,” “botanical drug,” “botanical drug formula”); and (3) Pharmacological research (“mechanism,” “antidepressant,” “clinical trial”). We provide an example of the adapted PubMed search string below, and we applied similar strategies—with appropriate translation and database-specific syntax—to all other databases.

PubMed Search String (Example):

(“Depression”[Mesh] OR “Depressive Disorder”[Mesh] OR depress*[tiab] OR “major depressive disorder”[tiab]) AND (“Medicine, Chinese Traditional”[Mesh] OR “traditional Chinese medicine”[tiab] OR “botanical drug*”[tiab]) AND (“Antidepressive Agents”[Mesh] OR pharmacolog*[tiab] OR mechanism*[tiab] OR antidepress*[tiab]).

### 3.2 Study selection and eligibility criteria

The study selection followed a predefined, two-stage screening process. After removing duplicates, two reviewers independently screened titles and abstracts for relevance. Potentially eligible studies then underwent full-text assessment against the following criteria.

#### 3.2.1 Inclusion criteria

##### 3.2.1.1 Study design

Original preclinical (*in vivo*, *in vitro*) or clinical research (randomized controlled trials, observational studies) published in peer-reviewed journals.

##### 3.2.1.2 Focus

Primary investigation of depression or validated depressive-like behaviors.

##### 3.2.1.3 Intervention

Any TCM intervention, including single botanical drugs, classic/modified botanical drug formulae, or their purified metabolites.

##### 3.2.1.4 Reporting

Studies reporting sufficient detail on the intervention (e.g., dosage, extract type), experimental model, and outcomes (behavioral or mechanistic).

#### 3.2.2 Exclusion criteria

Reviews, meta-analyses, editorials, case reports, and conference abstracts.

Studies where depression was a secondary outcome or not rigorously assessed.

Studies with insufficient pharmacological detail (e.g., missing dosage, uncharacterized extract).

Publications in languages other than English or Chinese.

### 3.3 Data extraction and critical appraisal

Data from included studies were extracted systematically, capturing: (1) TCM intervention with full taxonomic name where applicable; (2) Experimental model details (animal model, cell line, clinical design); (3) Treatment protocol (dose, route, duration, controls); (4) Key efficacy outcomes; and (5) Proposed mechanisms of action.

The methodological rigor of each study was critically appraised. For preclinical studies, we assessed aspects such as the use of positive/negative controls, dose-response relationships, and model validity. For clinical trials, we evaluated study design elements including randomization, blinding, and sample size.

### 3.4 Data synthesis

Given the heterogeneity in interventions and outcomes, we employed a narrative synthesis approach. We organized the evidence first by the type of TCM intervention (single botanical drugs, formulae, metabolites). Then we discussed thematically according to the primary mechanistic pathways involved (e.g., monoaminergic modulation, HPA axis regulation, neuroinflammation, neuroprotection). This synthesis not only summarizes findings but also explicitly highlights consistencies, contradictions, and gaps in the literature, forming the basis for the critical discussion in subsequent sections.

## 4 Pathogenesis of depression

The complex etiology of depression is not fully elucidated, with several interconnected hypotheses proposed to explain its underlying mechanisms. Key contemporary frameworks include: the monoamine hypothesis, neuroendocrine (HPA axis) dysregulation, the neurotrophic hypothesis, neuroinflammation, the gut microbiota-gut-brain axis model, and the influence of gut microbiota imbalance (Zhou et al., 2024; Troubat et al., 2021; Mikulska et al., 2021). These biological perspectives are further modulated and shaped by socio-environmental and genetic factors (Kubon et al., 2021).

### 4.1 The monoaminergic neurotransmitter hypothesis

The monoamine hypothesis proposes that a deficit in monoaminergic neurotransmission, primarily involving serotonin (*5-HT*), norepinephrine (*NE*), and dopamine (*DA*), is central to the pathophysiology of depression (Bhatt et al., 2021). This model successfully rationalized the mechanism of early antidepressant drugs, which act to increase synaptic levels of these neurotransmitters. However, the hypothesis has significant limitations. The therapeutic effects of such drugs typically manifest after a delay of several weeks despite their rapid biochemical action, and a substantial proportion of patients do not respond adequately. This temporal and efficacy disconnect suggests that monoamine dysregulation is likely a metabolite of a more complex, downstream pathological cascade rather than the sole causative factor (Spellman and Liston, 2020; Petra et al., 2015).

### 4.2 The hypothesis of immune responses and neuroinflammation

AA bidirectional link between depression and systemic inflammation is well-established. Pro-inflammatory cytokines can access the brain and contribute to depressive symptomatology, while stress and depressive states can potentiate inflammatory responses, potentially influencing antidepressant treatment efficacy (Beurel et al., 2020). Chronic stress activates the hypothalamic-pituitary-adrenal *(HPA)* axis and the sympathetic nervous system, promoting a peripheral pro-inflammatory state (Halaris, 2019). Within the central nervous system *(CNS)*, microglia—the resident immune cells—become activated and release pro-inflammatory factors, including interleukin-1β *(IL-1β)*, *IL-6*, and tumor necrosis factor-α *(TNF-α)*. This neuroinflammatory milieu, particularly in limbic regions like the hippocampus, can lead to neuronal damage and reduced neurogenesis, thereby contributing to the development of depressive symptoms (Jia et al., 2021).

### 4.3 The neurotrophic developmental hypothesis

The neurotrophic hypothesis centers on brain-derived neurotrophic factor (*BDNF*), a key regulator of neuronal survival, growth, and synaptic plasticity. *BDNF* exerts its effects primarily by binding to its high-affinity receptor, tropomyosin-related kinase B *(TrkB)*, which activates intracellular signaling pathways such as phosphatidylinositol 3-kinase *(PI3K)*/Protein Kinase B *(Akt)* and mitogen-activated protein kinase *(MAPK)*/extracellular signal-regulated kinase *(ERK)*, crucial for neuronal function (Castrén and Monteggia, 2021). Depression often reduces *BDNF* levels in critical brain regions, such as the hippocampus and prefrontal cortex. A core action of many antidepressants is the restoration of *BDNF* signaling, which promotes synaptic plasticity and neurogenesis—processes believed to be integral to mood recovery (Rahmani et al., 2020; Malhi and Mann, 2018).

### 4.4 The microbiota-gut-brain axis hypothesis

The gut-brain axis hypothesis posits that bidirectional communication between the gut microbiota and the central nervous system influences mood and behavior. Gut microbiota-derived metabolites, such as short-chain fatty acids, can signal to the brain via immune, endocrine, and neural pathways (e.g., the vagus nerve). Furthermore, the gut microbiota is involved in the synthesis and metabolism of key neurotransmitters, including serotonin (*5-HT*) and γ-aminobutyric acid (*GABA*) (Peirce and Alviña, 2019). Alterations in gut microbial composition (dysbiosis) are linked directly to depression. They may contribute to its pathophysiology by promoting systemic inflammation, impairing intestinal barrier function, and modulating tryptophan metabolism—the precursor of serotonin (Simpson et al., 2021; Rieder et al., 2017).

### 4.5 The hypothalamic-pituitary-adrenal *(HPA)* axis hypothesis

The hypothalamic-pituitary-adrenal *(HPA)* axis is the body’s primary neuroendocrine stress response system. In depression, this axis frequently becomes dysregulated. Although the fundamental sequence of corticotropin-releasing hormone (*CRH*) and adrenocorticotropic hormone (*ACTH*) leading to glucocorticoid (e.g., cortisol) release remains intact, the critical negative feedback mechanism is often impaired (Bingham et al., 2021). This results in sustained HPA axis hyperactivity and elevated glucocorticoid levels, which can exert detrimental effects on limbic brain structures, such as the hippocampus, and contribute to the pathogenesis of depressive symptoms (Kinlein et al., 2019).

### 4.6 The gene-environment-epigenetic interaction hypothesis

Depression susceptibility arises from complex interactions between genetic predisposition and environmental exposures, particularly early-life stress (ELS). ELS can induce stable epigenetic modifications—such as DNA methylation, histone modifications, and altered non-coding RNA expression—that regulate gene expression without altering the DNA sequence itself, providing a mechanistic link between experience and long-term biological vulnerability (Juruena et al., 2021). Researchers have identified stress-related epigenetic alterations in genes involved in key depression-associated pathways, including HPA axis regulation (e.g., *NR3C1*, *FKBP5*), serotonin signaling (e.g., *SLC6A4*), and neurotrophic support (Park et al., 2019). These findings underscore the role of epigenetic dysregulation in depression and highlight potential targets for novel therapeutic strategies.

## 5 From “liver qi stagnation” to modern neurobiology: decoding the pathophysiological basis of depression in TCM

A growing body of modern neuroscientific evidence provides empirical support for the core TCM pathogenesis of “Liver Qi Stagnation” (a syndrome characterized by stagnation of liver qi flow, manifesting as emotional distress and irritability), directly linking it to specific neurobiological abnormalities observed in depression. Contemporary research further connects the broader TCM etiology of “Qi Stagnation” (impaired flow of vital energy, associated with systemic dysfunction) to multi-scale pathological mechanisms in depression.

At the circuit level, impaired medial prefrontal cortex (*mPFC*)→paraventricular thalamus (*pPVT*) pathway activity, which can causally induce or rescue social deficits, reflects the functional blockade described as “Liver Qi Stagnation Disturbing the Mind” (Yamamuro et al., 2020). Gut microbiota dysbiosis in patients, including reduced Faecalibacteriumcorrelated with symptom severity, aligns with the concept of “Liver Qi Invading the Spleen” (where stagnant liver qi disrupts spleen function) (Jiang et al., 2015). Depression is also characterized by impaired neural plasticity—reduced neurogenesis, synaptic dysfunction, and downregulated *BDN*F signaling—which corresponds to “Qi Stagnation and Blood Stasis,” while TCM interventions can reverse these deficits (Xia et al., 2021; Zeng et al., 2025; Hirshler and Doron, 2017). The hippocampus, as one of the most extensively studied brain regions in depression, exhibits marked volume atrophy in affected patients, which significantly compromises neuroplasticity. Notably, traditional Chinese medicine (TCM) interventions can mitigate such impairments. For instance, Xiaoyao San, a classic TCM formula clinically employed for depression with the effects of soothing the liver and invigorating the spleen, may exert its antidepressant action by modulating hippocampal neuroplasticity (Zhang W. J. et al., 2024). Furthermore, nutrient-metabolic influences on epigenetic regulation (e.g., vitamin B12 affecting transthyretin and DNA methylation) relate to “Qi Stagnation Transforming into Fire” (Stein et al., 2025), Additionally, stress-induced compromise of blood-brain barrier (BBB) integrity offers a mechanistic basis for “Disordered Qi Assailing the Brain Collaterals” (Dion-Albert et al., 2022).

In summary, the TCM framework of the “Qi Stagnation” conceptually integrates a network of systemic dysfunctions spanning neural circuits, gut-brain axis, neuroplasticity, epigenetics, and vascular integrity. This multi-level correspondence not only grounds the TCM construct in modern biology but also explains how TCM therapies, through multi-target modulation of this network, can effectively address the systemic nature of depression (Xia et al., 2021; Zeng et al., 2025; Hirshler and Doron, 2017).

Given the close association between “Qi Stagnation” and the aforementioned pathological alterations, liver-soothing and qi-regulating TCM botanical drug formulas may exert therapeutic effects by targeting these pathways, which we will explore in detail in the following section (Xia et al., 2021).

## 6 Treatment of depression with traditional Chinese Medicine

The treatment of depression with Traditional Chinese Medicine (TCM) follows its core principles of syndrome differentiation and treatment individualization. This approach aims to alleviate symptoms by rebalancing the functions of the zang-fu organs (e.g., liver, spleen, heart) and regulating the flow of qi (Si Fuchun and Liu, 2011).

Within the TCM diagnostic framework, depression falls under the category of “Yu Syndrome” (郁证). This condition primarily features stagnation, with the liver’s failure to ensure the free flow of qi acting as the central pathological mechanism. This dysfunction subsequently leads to disharmony of other organs—most commonly the spleen (affecting digestion and overthinking) and the heart (affecting spirit and sleep)—and impairs the circulation of both qiand blood (Zhang X. Z. S. et al., 2024). Classical medical texts, such as the Huangdi Neijing (《黄帝内经》), emphasize the critical importance of unobstructed qi movement for health. Later works, including the Zhubing Yuanhou Lun (《诸病源候论》), further elaborate on diseases stemming from emotional disturbances and stagnation. Therefore, the core therapeutic principle is to soothe the liver to promote qiflow, resolve stagnation, and restore harmony among the affected zang-fuorgans.

Building upon this theoretical foundation, the following sections synthesize contemporary research. They integrate the traditional applications of TCM interventions with modern pharmacological insights, reviewing the evidence for single botanical drugs, metabolite formulae, and purified metabolites that exhibit antidepressant effects through mechanisms such as neurotransmitter modulation, anti-inflammatory actions, and the upregulation of neurotrophic factors (Dong and Huang, 2022).

### 6.1 Single botanical drug

A review of clinical practice and modern pharmacopoeias identifies several botanical drugs frequently employed in TCM for managing depression (“Yu Syndrome”). Representative examples include *Poria cocos*(Schw.) Wolf [Polyporaceae; Poria], *Angelica sinensis*(Oliv.) Diels [Apiaceae; Angelicae Sinensis Radix], *Ziziphus jujubaMill*. var. spinosa(Bunge) Hu ex H.F.Chou [Rhamnaceae; Ziziphi Spinosae Semen], *Polygala tenuifolia*Willd. [Polygalaceae; Polygalae Radix], and *Bupleurum chinense*DC. [Apiaceae; Bupleuri Radix]. The following discussion focuses on selected drugs for which robust preclinical evidence elucidates specific mechanisms of action.

#### 6.1.1 *Nardostachys jatamansi* (gan song)

*Nardostachys jatamans*i(D.Don) DC. [Caprifoliaceae; Nardostachydis Radix et Rhizoma], known as Gansong, is traditionally used to regulate qiand relieve pain. Preclinical studies substantiate its antidepressant potential, primarily linked to modulation of the serotonergic system. The total methanolic extract of N. jatamansi (58–255 mg/kg) and a purified water-soluble fraction (NJFr.01, 73–160 mg/kg) significantly reduced immobility time in the tail suspension test (TST) in mice, without affecting locomotor activity in the open field test (OFT). This behavioral improvement correlated with a marked enhancement of serotonin transporter (*SERT*) function, suggesting a mechanism distinct from classic *SERT* inhibition (Li R. et al., 2021). These findings translate the traditional application of Gansong for “qistagnation” into a plausible modern pharmacological action, positioning it as a source of novel *SERT*-modulating agents. Further research is needed to identify the specific active metabolites responsible and to evaluate efficacy in chronic stress models.

#### 6.1.2 *Paeonia lactiflora* (Baishao)

*Paeonia lactiflora*Pall. [Paeoniaceae; Paeoniae Radix Alba], or Baishao, is a fundamental botanical drug in TCM for nourishing blood and soothing the liver. Its antidepressant properties have been extensively studied, with paeoniflorin as a key bioactive metabolite. In a chronic unpredictable mild stress (CUMS) mouse model, intraperitoneal administration of paeoniflorin (20 mg/kg) ameliorated depressive-like behaviors and promoted hippocampal neurogenesis. In the rat forced swimming test (FST), oral paeoniflorin (10 mg/kg) significantly reduced immobility time. The mechanism of action is multi-target, involving modulation of the *MAPK/ERK* signaling pathway, upregulation of *BDNF*, normalization of HPA axis hyperactivity, and suppression of neuroinflammation—consistent with a holistic reversal of the pathophysiological network associated with depression (Wang et al., 2021). While these preclinical results are compelling, they are derived primarily from acute or sub-chronic models. The translational promise of paeoniflorin requires validation in long-term studies and, crucially, in well-designed clinical trials to establish its human efficacy, optimal dosage, and safety profile (Wang X. L. et al., 2022).

#### 6.1.3 *Panax ginseng* (ren shen)

*Panax ginseng*C.A.Mey. [Araliaceae; Ginseng radix et rhizoma], known as ginseng, is a highly valued botanical drug in TCM. Ginsenosides, a class of antioxidant triterpene saponin metabolites present throughout the plant, **primarily underpin** its neuroprotective and adaptogenic properties (Attele et al., 1999; Shi et al., 2019). Research indicates that ginseng can modulate stress-induced immune and hormonal responses, helping to maintain physiological homeostasis. Evidence demonstrates its efficacy in regulating the HPA axis, correcting endocrine imbalances, alleviating depressive behaviors, and reducing inflammatory states (Lee and Rhee, 2017; Mu and Ma, 2023).

#### 6.1.4 *Cyperus rotundus* (Xiang fu)

*Cyperus rotundus*L. [Cyperaceae; Cyperi rhizoma], or Cyperi Rhizoma, is a fundamental TCM botanical drug used to soothe liver disharmony, regulate qiflow, and relieve depression (Wang F. et al., 2022). Preclinical studies support its antidepressant potential. In a CUMS mouse model, the metabolite α-cyperone (0.5–1 mg/kg, i.g.) reduced immobility time in the TST and FST, an effect linked to modulation of the sirtuin 3 (*SIRT3)/NLRP3* signaling pathway. Furthermore, various extracts (ethanol, essential oil, aqueous) of C. rotundus (50–800 mg/kg) have shown efficacy in improving depression-like behaviors in both acute (TST, FST) and chronic stress models, likely through multi-target mechanisms involving monoamine neurotransmitter regulation and neuroprotection (Lu et al., 2022).

#### 6.1.5 *Poria cocos* (fu ling)

*Poria cocos*(Schw.) Wolf [Polyporaceae; *Poria cocos*], a medicinal mushroom, is used for its digestive and calming effects. Polysaccharides and triterpenes **underpin** its antidepressant properties. Acidic polysaccharides (0.1–0.5 g kg^-1^·d^-1^, p.o.) alleviate depressive behaviors and hippocampal inflammation in CUMS rats (Chen et al., 2021). Triterpene metabolites, such as poricoic acid B, demonstrate multi-target actions: they regulate neurotransmitters (5-HT, DA), suppress HPA axis activity and the *NLRP3* inflammasome pathway (reducing neuroinflammation and pyroptosis), protect hippocampal and prefrontal cortical neurons, and modulate the gut microbiota (Xiang, 2022). Network pharmacology suggests shared targets with known antidepressants, supporting P. cocostriterpenes as promising candidates (Zhang J. Y. et al., 2019).

#### 6.1.6 *Angelica sinensis* (dang gui)

*Angelica sinensis*(Oliv.) Diels [Apiaceae; Angelicae sinensis radix], known as Danggui, is traditionally used to nourish blood. Studies indicate antidepressant potential for its volatile oil and polysaccharides. In CUMS mice, *Angelica sinensis* volatile oil (15–60 mg/kg) improved depressive-like behaviors (increased locomotion, increased sucrose preference, reduced TST immobility), potentially by modulating brain 5-HT and DA levels and hippocampal AMPA receptor activity (Xie and Liang, 2024). Polysaccharides from Danggui upregulated tryptophan hydroxylase 1 (TPH1) mRNA expression (a key enzyme in 5-HT synthesis) and increased DA levels in CUMS mice, thereby ameliorating depressive symptoms (Wigner et al., 2018; Ding and Ge, 2021). These findings provide preliminary evidence for its antidepressant effects, warranting further investigation into the active metabolites and their mechanisms.

#### 6.1.7 *Ziziphus jujubavar .spinosa* (suan zao ren)

*Ziziphus jujubaMill. var. spinosa*(Bunge) Hu ex H.F.Chou [Rhamnaceae; Ziziphi spinosae semen], known as sour jujube seed, is used in TCM to calm the mind. Researchers attribute its antidepressant effects to metabolites such as saponins and flavonoids, which regulate neurotransmitters including 5-HT, DA, and NE, as well as GABA (Zuo Jun et al., 2017). Studies have shown that the saponin jujuboside A (JuA, 10–30 mg/kg) enhances hippocampal neuronal survival and increases levels of *BDNF, TrkB, 5-HT*, and dopamine, demonstrating antidepressant potential in preclinical models (Tao, 2023).

#### 6.1.8 *Polygala tenuifolia* (yuan zhi)

*Polygala tenuifolia*Willd. [Polygalaceae; Polygalae radix], or Yuanzhi, is traditionally used to tranquilize the mind and improve cognition. Its extracts (0.13–1 g/kg) and active metabolites (e.g., senegenin, 4–9 mg/kg) show antidepressant effects in chronic stress and behavioral despair models. Mechanisms include modulation of monoamine neurotransmitters (e.g., via 5-HT2A receptor), promotion of hippocampal neurogenesis, and activation of the *BDNF* pathway. Over 140 metabolites, including saponins, contribute to its neuroprotective, anti-inflammatory, and antioxidant activities (Zhao et al., 2020). The extract alleviates depressive behaviors by enhancing synaptic plasticity, hippocampal neurogenesis, and *BDNF* signaling, providing a preclinical basis for its development (Lacaille-Dubois et al., 2020; Jiang et al., 2021; Hu et al., 2010).

#### 6.1.9 *Bupleurum chinense* (chai hu)

*Bupleurum chinense* DC. [Apiaceae; Bupleuri radix], commonly known as Chaihu, is traditionally utilized in Traditional Chinese Medicine for soothing liver qi stagnation. Core bioactive metabolites, including saikosaponins A, B2, D, and total saikosaponins, primarily mediate its antidepressant effects. Researchers have validated these metabolites in various depressive animal models, such as CUMS and chronic social defeat stress (CSDS).

Experimental designs typically employ low-, medium-, and high-dose gradients (e.g., 25, 50, and 100 mg/kg for saikosaponin A). Results demonstrate that these bioactive metabolites significantly elevate the expression levels of phosphorylated cyclic adenosine monophosphate response element-binding protein *(p-CREB)*, *BDNF*, and the anti-apoptotic protein B-Cell Lymphoma 2 (*Bcl-2*) in the hippocampus of model animals. Concurrently, they downregulate the expression of the pro-apoptotic proteins *Bcl-2*-Associated X Protein (*Bax*) and Cysteine-Aspartic Acid Protease 3 (*Caspase-3*).

These mechanisms suggest that *Bupleurum chinense* and its bioactive metabolites exert antidepressant effects through core pathways that upregulate *BDNF* signaling and inhibit neuronal apoptosis, providing robust experimental evidence for its clinical application (Chen et al., 2024).

### 6.2 Traditional Chinese Medicine formulae

Clinicians widely use TCM formulae to treat depression. Based on clinical guidelines and empirical research, this section analyzes several classic and commonly adapted formulations. The following discussion provides a detailed examination of their clinical applications and therapeutic mechanisms (Table 1).

**TABLE 1:** Therapeutic profiles of representative TCM formulae in depression research.

Formula Drug	Animals	Modeling	Administration	Dosage	Behavioral tests	Key detection	TCM basis	Mechanism	Conclusion	References
1. Jieyu Pills (JYP)	None (clinical human study)	None (naturally-occurring PSD patients with liver qi stagnation)	Oral	4 g per time, 3 times a day, for 4 consecutive weeks	None	HAMD score, TCM syndrome score, serum NE/DA/5-HT	Soothe the liver and relieve stagnation, nourish the heart and tranquilize the mind for liver qi stagnationSoothes liver, regulates Qi, alleviates depression, calms spirit	Elevate NE/DA/5-HT, activate monoamine neurotransmitter system	Combined use improves efficacy, relieves depression, no obvious adverse reactions, safe and effective	Jun (2022)
2. Zhi-Zi-Chi Decoction (ZZCD)	Male Sprague-Dawley rats (180–220 g, n = 8/group)	CUMS + single housing for 7 weeks	Oral gavage	5 g raw botanical drug/kg/day (last 2 weeks)	SPT, FST, TST, body weight	Brain Reactive Oxygen Species(ROS), GSH/GSSG, SOD, Glutathione Reductase(GR), Glutathione Peroxidase (GPx); metabolomics	From Shang Han Lun; clears heat and resolves irritability	Reduces cerebral oxidative stress via regulating GSH/GSSG pathway and amino acid metabolism	ZZCD exerts antidepressant effects by ameliorating oxidative damage in the brain	Zhang Y. et al. (2022)
3. Jiaotai Pill (JTP)	Healthy male SD rats (140–160 g)	CUMS (35 days, random stressors)	Oral (i.g.)	Crude drug 0.75, 1.50, 3.00 g/kg (calculated based on 33.4% extraction yield)	OFT (crossings and rearings in 5 min)	(1) Body weight change (2) Sucrose preference (3) Brain NE/5-HT/5-Hydroxyindoleacetic Acid (5-HIAA)levels High-Performance Liquid Chromatography (HPLC)	Regulates “Heart-Kidney non-interaction” (Xin-Shen Bu Jiao, classical theory from Han’s Medical Book and Four Branches of Simplified Formulas)	Increases NE/5-HT, decreases 5-HIAA	ignificant antidepressant effects, improves depression-related behaviors and neurotransmitter imbalance	Yang et al. (2016), Dai Guo-Liang et al. (2021)
4. Xiaoyao Pill (XYP)	Female Kunming mice (18–23 g)	Sleep deprivation via modified multiple platform method (7 days)	Oral gavage	Low: 0.32 mg/kg; High: 0.64 mg/kg (2 weeks)	Morris Water Maze (MWM), OFT, SPT, TST, irritability scoring	Neurotransmitter levels (DA, NE, 5-HT) in hippocampus and prefrontal cortex (HPLC-ECD), Cellular Fos Oncogene(c-Fos) expression (immunohistochemistry), and brain histopathology (H&E staining)	Addresses Liver-Qi Stagnation and Spleen Deficiency functions to soothe liver, tonify spleen, nourish blood	Attenuates affective dysregulation via neuroprotection and monoamine modulation	Xiaoyao Pill alleviates sleep deprivation-induced affective dysregulation by repairing brain injury and restoring monoamine neurotransmitter levels.Low dose showed better overall effects	Li et al. (2022)
5. Chai gui Granules (CGKL)	Male Sprague Dawley rats, 8 weeks old, weighing (200 ± 20) g	CUMS, lasting for 7 weeks	Oral gavage (i.g.)	8.3 g/kg, once daily for 4 consecutive weeks	Body weight change, SPT, OFT, FST	Gut microbiota; arginine metabolites; hippocampal Ornithine Decarboxylase (ODC)/Spermidine/Spermine N1-Acetyltransferase 1 (SAT1)/Agmatinase (AGMAT)	Derived from Xiaoyao San; targets liver-spleen disharmony	Regulates gut microbiota and arginine metabolism pathway	CGKL exerts antidepressant effects via gut microbiota–arginine metabolism axis	Wang et al. (2024)
6. Guipi Decoction (GPD)	2-month-old female SD rats (160–180 g)	CUMS + solitary housing, 28 d	Gastric gavage	12.5 g/kg, once daily for 28 consecutive days	OFT, SPT, Forced swimming test, Emotional behavior scoring	Body weight gain; Hippocampal 5-HT, NE, DA levels (detected by ELISA)	Yu Syndrome (heart-spleen deficiency); Tonify qi/blood, invigorate spleen, nourish heart	Elevates the levels of 5-HT, NE and DA (monoamine neurotransmitters) in the hippocampus of depressed rats, ameliorates the imbalance of cerebral monoamine neurotransmitters	Ameliorate depressive behaviors, reverse reduced monoamine neurotransmitters	Dong et al. (2017)
7. Huanglian Ejiao Decoction (HLED)	Male Sprague-Dawley (SD) rats (n = 55, 180–200 g, SPF-grade)	Chronic sleep deprivation (12 h/day for 21 days) using a modified water-platform apparatus	Intragastric gavage (0.01 mL/g, once daily for 14 days)	HLED (low: 1.925, medium: 3.85, high: 7.7 g/kg); Eszopiclone (0.1 mg/kg)	Pentobarbital-induced sleep test, OFT, SPT	Serum/hippocampus: 5-HT, 5-HIAA, TPH, MAO-A (ELISA); Hypothalamus: 5-HT1AR/2AR mRNA (qPCR); Gut microbiota (16S rRNA sequencing)	Indicated for “Yin deficiency with effulgent fire” and “heart-kidney non-interaction” insomnia (per Shang Han Lun)	Modulated 5-HT system (increased 5-HT/5-HT1AR, decreased 5-HT2AR) and gut microbiota (higher Firmicutes/Bacteroidetes ratio	HLED ameliorated sleep deprivation-induced insomnia and depressive-like behaviors	Qiong (2023)
9. Peiyuan Jieyu Decoction (PYJYD)	SPF male ICR mice, 22–25 g	① Reserpine (2.5 mg/kg, i.p.); ② Acute stress: forced swimming; ③ Chronic stress: 14 days restraint + noise	Gastric gavage, once daily (7/14 days for stress models, simultaneous for reserpine model)	4.60/2.30/1.15 g/kg (H/M/L)	① Reserpine model: temperature, ptosis, akinesia; ② Stress models: forc	Brain: 5-HT, NA, Indoleamine 2,3-Dioxygenase (IDO) (ELISA); Chronic stress: IDO mRNA (qPCR)	Soothe liver, tonify primordial qi, enrich essence (based on liver stagnation)	Elevate brain 5-HT/NA, reduce IDO activity/mRNA, antagonize reserpine-induced abnormalities	Ameliorates depressive symptoms via regulating 5-HT/NA and inhibiting IDO	Liu et al. (2017)
9. Danzhi Xiaoyao San (DXS)	Male Sprague-Dawley rats, 200 ± 20 g	CUMS for 21 days with randomized exposure to 9 types of mild stressors	Oral gavage, twice daily	4 g/kg/day (aqueous extract)	Body weight, open-field test (crossing and rearing numbers), sucrose preference, food consumption	Serum *TNF-α, IL-6;* hippocampal IDO protein/mRNA, Tryptophan (Trp), Kynurenine (Kyn), *5-HT, Kyn*/Trp ratio	Soothes the liver, clears heat, and nourishes blood; used for emotional disorders due to “liver qi stagnation with heat transformation,” consistent with CUMS model pathogenesis	Suppresses inflammation → downregulates hippocampal IDO → shifts tryptophan metabolism from Kyn to 5-HT pathway	DXS ameliorates depressive-like behavior by regulating the “inflammation–tryptophan metabolism” axis and increasing 5-HT levels	Zhu et al. (2015)
10. Kaixinjieyu (KJ)	SD rats, male, 250 ± 10 g	LBCCA + CUMS for 21 days	Oral gavage	1.8, 0.9, 0.45 g/kg/d (high/medium effective)	Improved sucrose preference, movement and rearing	*BDNF, TrkB*, Tissue Plasminogen Activator (t-PA), Plasminogen Activator Inhibitor-1 (PAI-1), Matrix Metalloproteinase 2 (MMP-2), Zona Occludens 1 (ZO-1), occludin, claudin-5, Terminal Deoxynucleotidyl Transferase dUTP Nick-End Labeling (TUNEL), Cerebral Blood Flow MDA (CBF)	Tonifies Qi and relieves depression	Promotes neurogenesis, strengthens BBB, balances fibrinolysis	KJ improves depression and cerebral hypoperfusion via NVU repair	Pan et al. (2015)
11. Zishui Qinggan Decoction (ZSQGD)	Male C57BL/6 mice (6–8 weeks, 20 ± 2 g)	21-day chronic restraint stress (CRS), validated by sucrose preference <75%	Oral gavage (0.4 mL/20 g)	ZSQGD low/medium/high: 8.835/17.670/35.340 g/kg	Sucrose preference, tail suspension, forced swimming tests	Serum: Superoxide Dismutase (SOD), Malondialdehyde (MDA), *5-HT, TNF-α, IL-1β*Hippocampus: HE staining, *BDNF/TNF-α/IL-1β mRNA, ERK/GSK3β/CREB/BDNF* proteins	Nourishes kidney yin, clears liver fire; addresses “liver depression with kidney yin deficiency” pattern	Activated *ERK/GSK3β/CREB/BDNF* pathway, increased *p-ERK1/2, p-GSK3β, CREB,* and *BDNF,* decreased *TNF-α* and *IL-1β*, and enhanced *SOD* activity while reducing *MDA*.	Optimal efficacy at 17.670 g/kg, comparable to fluoxetine	Cao et al. (2024)
12. Shenhu Wendan Decoction(SHWDD)	Male ICR mice (behavioral despair model); Male Wistar rats (CUMS model)	Mice: TST and FST; Rats: CUMS with sucrose preference screening	Intragastric gavage (mice: 16 days; rats: 15 days)	Mice: 23.52/11.76/5.88 g/kg Rats: 16.48/8.24/4.12 g/kg	Mice: TST + FST Rats: SPT+ body weight measurement + OFT	*5-HT/DA/NE; IL-1β/IL-6/TNF-α/IL-4/IL-10; p-ERK/CREB/BDNF/TrkB*	Liver-soothing and phlegm-resolving effects (modified Wendan Decoction)	Upregulated monoamine neurotransmitters, modulated inflammation, activated *ERK-CREB-BDNF* pathway	Improved depressive behaviors via neurotransmitter regulation and *ERK* pathway activation	Ying (2019)
13. Gan-Mai-Da-Zao Decoction, (GMDZ)	Anxiety models in mice: EPM test and LDB test	Normal, non-stressed mice	Oral (p.o.), once daily for 7 days	1, 2, and 4 g/kg per day for 7 consecutive days. Behavioral tests were conducted 60 min after the final administration	Elevated plus maze (EPM), light/dark box (LDB), marble burying (MB), open field (OF), rota-rod	Increased time in open arms (EPM), increased time in light box (LDB), reduced marble burying; no effect on locomotor activity or motor coordination	Used in traditional Chinese medicine for “Zang Zao syndrome” characterized by anxiety-like emotional disturbances	Involvement of GABAergic and 5-HT1A receptor systems	GMDZ produces anxiolytic-like effects without sedation or motor impairment	Chen et al. (2018)
14. Modified Sinisan	Male Wistar rats (SPF, 21days, 30–36 g)	CUMS (random stressors: restraint, wet cage, shock, etc.)	Daily oral gavage (before stress)	1.69 g/mL	OFT (activity, center time)	Hippocampal *5-HT1AR* expression	Regulates “Liver Qi Stagnation” in stress	Increased hippocampal 5-HT1AR reduces depressive behaviors	Improves depression-like behavior via 5-HT1AR	Shi et al. (2018)
15. Morinda officinalis oligosaccharide capsules (active metabolite: Morinda oligosaccharides)	86 patients with depression (clinical trial, no animal studies)	Not applicable (clinical study; patients met Chinese Classification of Mental Disorders 3rd Edition (CCMD-3) diagnostic criteria for depression and TCM kidney deficiency syndrome criteria)	Oral administration, 0.3 g per dose, twice daily (bid)	0.3 g per dose, twice daily (total 0.6 g/day)	Hamilton Depression Scale (HAMD-17), Hamilton Anxiety Scale (HAMA), TCM Kidney Deficiency Scale	Serum NGF, *5-HT, BDNF*, cortisol (Cor) levels	Regulates emotion, calms spirit, tonifies kidney and improves cognition”; alleviates kidney deficiency syndrome (core symptoms: depressed mood, insomnia, etc.)	Potential antidepressant effects via decreasing serum NGF and Cor while increasing *5-HT* and *BDNF* expression	Total effective rate 95.35%; significantly improved depressive symptoms with good safety profile (adverse reaction rate 16.28%)	Song et al. (2024)
16. Zhimu-Baihe saponins combination (AL)	Male Sprague-Dawley rats (180–200 g)	CUMS for 6 weeks with various random stressors	Oral gavage	48 mg/kg (combined saponins from Anemarrhena and Lilium)	FST, SPT	Levels of *5-HT, NE*, and *DA* in the hippocampus; Hippocampal metabonomic profiling	Derived from the classic formula “Baihe Zhimu Tang” used for treating “lily disease,” whose symptoms highly resemble those of depression	Regulation of monoamine neurotransmitter synthesis (especially 5-HT and NE), and restoration of amino acid, fatty acid, and phospholipid metabolism	AL exerts significant antidepressant-like effects, reverses CUMS-induced behavioral and biochemical abnormalities, and shows synergistic effects compared to individual metabolites	Du et al. (2016)
17. Roukou Wuwei Pills (RWP)	Female SD rats, 8–10 weeks old, 180–220 g	Female SD rats (180–220 g)	Oral gavage	8 g/mL suspension, 1 mL/100 g body weight (actual dose ≈80 mg/kg)	SPT, OFT (horizontal/vertical scores), forced swimming test	Hippocampal *5-HT, DA*, and *BDNF* levels (ELISA)	Classical Mongolian medicine for treating “Heyi” disease (regulates Qi movement)	Upregulates hippocampal *5-HT, DA,* and *BDNF* expression	Significantly improved behavioral indicators and neurotransmitter levels in PPD rats (optimal efficacy)	Xiao-Jing and Yong-Le (2023)
18. Modified Wendan Decoction (MWDD)	SPF-grade male SD rats, body weight 180–200 g	CUMS, 21 days of random stressors including food/water deprivation, cold swimming, tail suspension, etc.)	Intragastric gavage	12 g/kg (once daily for 35 days)	OFT (recorded horizontal movement, vertical movement, and total distance traveled)	*5-HT1A* protein expression in hippocampal CA3 region, amygdala, prefrontal cortex, and hypothalamus (Western blot)	Regulates qi and relieves depression, harmonizes cold-warm properties, resolves phlegm, and balances emotions (based on TCM theory of ascending-descending regulation)	Downregulates 5-HT1A protein expression in the hippocampal CA3 region	Modified Wendan Decoction exerts antidepressant effects by reducing *5-HT1A* protein expression in the hippocampal CA3 region	Zhang et al. (2023)
20. Dang gui-Shao yao-San (DSS)	Male ICR mice, weighing 20–25 g	(1) Reserpine-induced ptosis; (2) Chronic unpredictable stress (CUS)	Intragastric gavage	Low dose: 1.5 g/kg; High dose: 3 g/kg	(1) Ptosis score assessment; (2) SPT	(1) Brain monoamine neurotransmitter levels (*NA, DA, 5-HT*); (2) Serum MDA level and SOD activity	Managing circulation, supporting hepatic function, fortifying the digestive system, and dispelling humidity-related conditions	(1) Modulation of central monoaminergic systems (especially increasing NA and DA); (2) Reduction of oxidative stress	DSS exerts antidepressant-like effects, likely mediated by regulating monoamine neurotransmitters and inhibiting oxidative stress	Huang et al. (2012)
21. Li huan Jie yu Capsule (LJC)	Wistar rats (SPF grade), male, body weight 180–220 g	Single-cage housing + CUMS (8 alternating stressors for 21 days)	Intragastric gavage (10 mL/kg), daily at 9:00–10:00 AM	Low-dose group: 1.18 g/kg; High-dose group: 2.35 g/kg	Forced swimming test (5min immobility time); TST (4min immobility time)	Serum *NE, DA, 5-HT, CORT* levels (ELISA); Hippocampal *BDNF* protein expression (Western blot)	Soothing liver and relieving depression (Tribulus for liver regulation, Albizia for calming), resolving phlegm and opening orifices (Acorus), harmonizing effects (Glycyrrhiza)	ncreases monoamine neurotransmitters (*NE/DA/5-HT*), inhibits HPA axis (reduces CORT), upregulates hippocampal *BDNF* expression	Significant antidepressant effects, improves behavioral indicators and neurochemical abnormalities	Liu et al. (2016)
22. Xiao Bu xin Decoction total flavonoids (XBXT-2)	Male Wistar rats, SPF grade, 220–240 g	Chronic unpredictable stress (CUS) model with random stressors (tail clamping, food deprivation, cold water swimming, etc.) for 35 consecutive days	Intragastric gavage (ig), once daily	100 mg kg^-1^	SPT, OFT (rearing counts, center zone latency), novelty suppressed feeding test	Gut microbiota: Firmicutes/Bacteroidetes ratio, Lactobacillaceae abundance Inflammatory markers: Hippocampus/colon *IBA-1, TLR-4, IL-1β, IL-6, TNF-α, IL-10*	Resolving blood stasis and depression, promoting blood circulation and removing blood stasis Regulation of gut microbiota and brain	Regulates gut microbiota (increases beneficial bacteria, reduces pathogens) and attenuates gut-brain axis immune-inflammatory response	XBXT-2 exerts antidepressant and anxiolytic effects by modulating the microbiota-immune-brain axis pathway	Huang et al. (2020)

#### 6.2.1 Chaihu Shugan San (Bupleurum liver-soothing powder; CSS)

Chaihu Shugan San (CSS) is a classic traditional Chinese medicine (TCM) formula for relieving liver qi stagnation. It comprises seven botanical drugs: *Bupleurum chinense* DC. [Apiaceae; Radix Bupleuri], *Citrus reticulata* Blanco [Rutaceae; Pericarpium Citri Reticulatae], *Ligusticum chuanxiong* Hort. [Apiaceae; Rhizoma Chuanxiong], Citrus aurantium L. [Rutaceae; Fructus Aurantii], *Paeonia lactiflora* Pall. [Paeoniaceae; Radix Paeoniae Alba], *Cyperus rotundus* L. [Cyperaceae; Rhizoma Cyperi], and Glycyrrhiza uralensis Fisch. ex DC. [Fabaceae; Radix et Rhizoma Glycyrrhizae] (Yao, 2023). CSS takes soothing the liver and regulating qi as its core compatibility effect and can alleviate depressive symptoms. Paeoniflorin (PF), a key bioactive metabolite of CSS, exerts an intervention effect on menopausal depression. After intragastric administration of 10 mg/kg PF to rats for 2 weeks, PF exerts antidepressant effects by upregulating the mRNA expression of 5-hydroxytryptamine 1A receptor (5-HT_1_A R) and downregulating the mRNA expression of 5-hydroxytryptamine 2A receptor (5-HT_2_A R) (Guo et al., 2022). The total glucosides of paeony (TGP) from *Paeonia lactiflora* Pall. inhibit monoamine oxidase (MAO) activity, thereby slowing the degradation of monoamine neurotransmitters (Zhang and Huang, 2013). Network pharmacology and experimental studies suggest that the *PI3K/AKT* signaling cascade is a key pathway, with CSS promoting hippocampal neurogenesis and improving sucrose preference in depressed mouse models (Zhang S. et al., 2021). A rat model of liver-qi stagnation syndrome was established via chronic restraint stress combined with isolated breeding. The rats were intragastrically administered CSS at a low dose (0.52 g/mL crude drug) or high dose (1.04 g/mL crude drug) once daily for 3 weeks. Detected by 18F-FDG and 11C-Raclopride MicroPET/CT, this metabolite formula bidirectionally regulated regional cerebral glucose metabolism and corrected abnormal dopamine D_2_ receptor (D_2_R) binding potential, ameliorating behavioral abnormalities, HPA axis dysfunction, immune suppression, and monoaminergic neurotransmitter imbalance in the model rats, with the low dose exhibiting superior efficacy (Wang et al., 2023).

#### 6.2.2 Yueju Pill (Yue ju wan)

Yueju Wan is a formula composed of five botanical drugs: Cyperi Rhizoma (*Cyperus rotundus*), Ligustici Rhizoma (Ligusticum chuanxiong), Gardeniae Fructus (Gardenia jasminoides), Atractylodis Macrocephalae Rhizoma (*Atractylodes macrocephala*), and Massa Fermentata, traditionally used to regulate qiand relieve depression (Zhao and Long, 2024). Its mechanisms involve modulating glutamate receptors and neurotrophic signaling. The formula stimulates NMDA and AMPA receptors and rapidly increases hippocampal *BDNF* levels, correcting disruptions in the *Akt-mTOR* pathway in the prefrontal cortex of chronically stressed animals (Ren and Chen, 2017). In 7- to 8-week-old male Kunming (KM) and ICR mice (using the inherent difference in drug response duration between the two strains as an internal model), a single intragastric administration of Yueju Wan ethanol extract (270 mg/kg, solvent: 0.5% Tween 80 in saline, stock concentration: 270 mg/mL) was performed. Behavioral tests and pathway inhibition experiments confirmed that this metabolite exerts rapid and long-lasting antidepressant effects (sustained for 5 days in KM mice and 1 day in ICR mice) by activating the *cAMP/PKA/CREB/BDNF* signaling pathway (persistently upregulating the expression of related proteins and mRNA in the hippocampus). Its action is independent of the mTOR pathway (Xue et al., 2016). In a social isolation-induced “qi stagnation” model (female C57BL/6 mice, 30 days of modeling), administration of Yueju Wan (1.8 g/kg/d, intragastrically for 2 weeks) significantly elevates levels of adenosine, tryptophan, 5-HT, and GABA, regulating neuroendocrine modulators and mitigating depressive behaviors (Wei, 2022).

#### 6.2.3 Kai-Xin-San (KXS)

Kai-Xin-San (KXS), first recorded in the Tang Dynasty, is a classic formula containing *Panax ginseng* C.A. Mey. [Araliaceae; Ginseng radix et rhizoma] and *Polygala tenuifolia* Willd. [Polygalaceae; Polygalae radix], *Poria cocos* (Schw.) Wolf [Polyporaceae; Poria], and *Acorus tatarinowii* Schott [Araceae; Acori tatarinowii rhizoma]. Clinicians **use** it to replenish qi, nourish the heart, and calm the spirit. In a CUMS mouse model, KXS was administered orally at doses of 3 and 10 g/kg/day for 7 consecutive days, which significantly increased sucrose preference, reduced immobility time in the TST and forced swimming test (FST), inhibited hippocampal microglial activation, and decreased the levels of pro-inflammatory cytokines (*IL-1β, IL-6, TNF-α*). These antidepressant-like effects are associated with inhibition of the Toll-Like Receptor 4 (*TLR4*)/Inhibitor of Nuclear Factor Kappa-B Kinase (IKK)/Nuclear Factor Kappa-B *(NF-κB*) signaling pathway, suggesting that the suppression of microglia-mediated neuroinflammation is a key underlying mechanism (Qu et al., 2021). According to network pharmacology and molecular docking analyses, active metabolites in Kaixinsan, such as Asarone and Pachymic acid, can effectively bind to the 5-HT receptor (*5-HTR*), whereas Ginsenoside Rg1, despite its antidepressant activity, failed to bind to *5-HTR* in molecular docking. Multiple metabolites collectively act on targets including dopamine receptors (*DRD*) and adrenergic receptors (*ADRA*), comprehensively modulating the monoaminergic neurotransmitter system and related signaling pathways—such as the serotonergic synapse, *PI3K-Akt*, and *MAPK* pathways—thereby contributing to the antidepressant effect (Du et al., 2022).

#### 6.2.4 Xiaoyaosan (Xiaoyao powder)

Xiaoyaosan, first documented in the Taiping Huimin Heji Ju Fang (Formulary of the Pharmacy of Peace and Benevolence), comprises eight botanical drugs: *Atractylodes macrocephala* Koidz. [Asteraceae; Rhizoma Atractylodis Macrocephalae], *Paeonia lactiflora* Pall. [Paeoniaceae; Radix Paeoniae Alba], *Angelica sinensis* (Oliv.) Diels [Apiaceae; Radix Angelicae Sinensis], *Bupleurum chinense* DC. [Apiaceae; Radix Bupleuri], *Poria cocos* (Schw.) Wolf [Polyporaceae; Poria], *Zingiber officinale* Roscoe [Zingiberaceae; Rhizoma Zingiberis Recens], *Mentha haplocalyx* Briq. [Lamiaceae; Herba Menthae Haplocalycis], and Glycyrrhiza uralensis Fisch. ex DC. [Fabaceae; Radix et Rhizoma Glycyrrhizae] (Meng and Zhou, 2025). Xiaoyaosan contains multiple bioactive constituents, including paeoniflorin and ferulic acid, the latter derived from Radix Angelicae Sinensis, one of the principal herbal metabolites in the formulation. These metabolites have been implicated in mediating antidepressant-like effects through modulation of neuroinflammation, monoaminergic neurotransmission, the HPA axis, and neurotrophic signaling pathways, as evidenced by preclinical studies (Chen J. et al., 2022).

### 6.3 Purified metabolites with antidepressant potential

#### 6.3.1 Ginsenosides (from *Panax ginseng*)

Ginsenosides, the primary bioactive metabolites of *Panax ginseng*, exhibit neuroprotective properties relevant to mood disorders (Table 2). Ginsenoside Rd shows anti-inflammatory, antioxidant, and anti-apoptotic activities (Chen Y. Y. et al., 2022). Ginsenoside Rg1 (20 mg/kg/day, i.p.) improved depressive-like behaviors in chronic stress and hippocampal injury models. It exerted its effects by upregulating the astrocytic gap junction protein connexin 43 (Cx43) and restoring intercellular communication, highlighting a novel glial-mediated mechanism (Lou et al., 2020). Further translational research is needed to evaluate its clinical potential.

**TABLE 2:** Therapeutic profiles of representative bioactive natural metabolites and single drugs derived from TCM in depression research.

Botanical drug	Animals	Modeling	Administration	Dosage	Behavioral tests	Key detection	TCM basis	Mechanism	Conclusion	References
1. Baihe extract*(Lilii Bulbus extract)*	C57BL/6J male mice (18–22 g, 9–12 weeks)	CUMS, 21 days	Oral gavage	Not specified (“appropriate concentration”)	OFT (horizontal crossings, rearing), FST (immobility time), SPT (anhedonia assessment)	*MYC, Iba1, TNF-α, IL-1β, IL-6,* neuronal damage/apoptosis	Sedation, Tranquilizing the Mind and Antidepressant Effect	Suppression of MYC expression reduces microglial activation and neuroinflammation	Baihe extract exerts antidepressant effects via MYC-mediated suppression of neuroinflammation	Dan et al. (2022) Fu et al. (2022)
2. Morinda officinalis (Bajitian) (*Morindae Officinalis Radix*)	SD rats (male, 8 weeks); ICR mice	CUMS (56 days); reserpine injection (0.5 mg/kg, i.p.).	Oral gavage	MOO: 50–100 mg/kg; PSA: 100 mg/kg	SPT, FST, TST	*5-HTP/5-HT* (feces, plasma, brain); TPH, 5-HTPDC, BH_4_, VB_6_; microbiota (16S)	Relieves depression, tonifies the kidney, and eliminates dampness	Enhances gut 5-HTP production via TPH upregulation and 5-HTPDC inhibition, resulting in increased brain 5-HT.	5-HTP/5-HT (feces, plasma, brain); TPH, 5-HTPDC, BH_4_, VB_6_; microbiota (16S)	Zhang et al. (2022b)
3. L-Menthol*(Mentha haplocalyxBriq.)*	C57BL/6 mice (Male, 8–10 weeks)	FST	Oral (i.g.)	256 μmol/kg	FST	Immobility time	Liver-calming and mood-regulation	Modulates GABAergic system	L-Menthol exhibits rapid-onset antidepressant-like effects	Yuan (2018)
4. *Milletia speciosaChamp* (MS)	Male Kunming mice (20–24 g)	CUMS for 35 days, including food/water deprivation, cold swim, cage tilt, reversed light/dark cycle, etc	Intragastric gavage (i.g.)	20.0 g/kg	SPT, Open-field test (crossing number, rearing times)	*5-HT, NE, BDNF* in brain; serum metabolites (e.g., tryptophan, lactate, choline) via ^1^H-NMR	Disperse (or Smooth) Liver Qi, Nourish and Protect the Liver	Regulates MAOA, MAOB, ACHE, IDO1, COMT; affects tryptophan metabolism, neurotransmitter synthesis, phospholipid metabolism	MS alleviates depressive behaviors and biochemical disorders via multi-target, multi-pathway regulation	Su et al. (2021)
5. Tanshinol Sodium from*(Salvia miltiorrhiza Bunge*)	Male C57 mice	CUMS for 6 weeks	Oral (i.g.), daily for 3 weeks	Low (10 mg/kg), Medium (20 mg/kg), High (40 mg/kg)	OFT, SPT, FST, TST	1. Plasma ACTH, CORT (ELISA) 2. Hippocampal 5-HT, NE, DA (HPLC) 3. Hippocampal *p-CREB, BDNF* (Western Blot)	Promotes blood flow and calming	Suppresses HPA axis hyperactivity, increases monoamine levels, activates *CREB/BDNF* pathway	TS improves depression by regulating HPA axis and neurotrophic pathways	Zhong et al. (2018)
6. *Atractylodes macrocephala* polysaccharide (AMP)	Male ICR mice (18–20 g)	CUMS for 60 days	Oral gavage (5 mL/kg/day)	Low (0.0625 g/kg), Medium (0.125 g/kg), High (0.250 g/kg)	SPT	1. Brain *5-HT* (ELISA) 2. Gut microbiota (16S rDNA) 3. Serum metabolomics Liquid Chromatography-Mass Spectrometry (LC-MS)	Traditionally used for “strengthening the spleen and calming the mind”	Regulates gut microbiota (increases Firmicutes/Bacteroidetes ratio) and metabolic pathways	AMP alleviates depression via gut-brain axis regulation	Wu et al. (2024)
7. Astragalus polysaccharide (APS)	Male KM mice (18–22 g)	CUMS for 56 days	Oral gavage	0.2 g/kg (equivalent to 27 g/kg crude botanical drug)	OFT, forced swimming test, TST, SPT	1. Brain 5-HT2. Plasma ACTH/CORT3. Hippocampal neuron morphology4. *ADCY6/PKA/CREB-1/BDNF* protein expression	Astragalus (“sweet, warm; enters Lung/Spleen meridians”) treats Qi-deficiency depression	Activates AC/cAMP/PKA pathway; inhibits HPA axis hyperactivity	APS alleviates depression by regulating neurotransmitters, HPA axis, and AC/cAMP/PKA signaling	Jia-lei et al. (2022)
8. Gastrodin (Gas), derived from *Gastrodia elata* *Blume*	Male db/db mice (7-week-old)	Spontaneous type 2 diabetes model due to leptin receptor mutation	Oral gavage	70 and 140 mg/kg, once daily for 12 weeks	Morris water maze (MWM), novel object recognition (NOR), FST	Blood glucose, lipid profile, insulin resistance, hippocampal pathology, ER stress- and *NLRP3*-related proteins	Gastrodia elata, calms liver and extinguishes wind, sedates and calms the spirit	Inhibition of ER stress and *NLRP3* inflammasome activation	Gas improves cognitive dysfunction and depressive-like behaviors via suppressing ER stress and *NLRP3* inflammation	Ye et al. (2018)
9. Polygonatum sibiricum polysaccharide (PSP)	Male C57BL/6 mice (3 months, 20–25 g)	LPS injection (2 mg/kg, i.p.) and CUMS for 35 days	Oral gavage (i.g.)	100, 200, 400 mg/kg (LPS model); 400 mg/kg (CUMS model)	TST, FST, SPT, OFT	*5-HT, CORT, SOD, MDA, IL-1β, TNF-α, p-ERK, NF-κB,* Glial Fibrillary Acidic Protein (*GFAP), p-Akt, p-mTOR*, *caspase-3*, Glutamate Receptor AMPA Subunit 1/2 (GluA1/2), Glutamate Receptor NMDA Subunit 2A/B (GluN2A/B), Nissl staining	Tonifies Qi, nourishes blood and yin, replenishes deficiencies of five zang organs, addressing root causes of depression in TCM theory	Reduces oxidative stress, inflammation, and HPA axis hyperactivity; protects synapses and neurons via *Akt/mTOR* and *ERK/NF-κB* pathways	PSP prevents depression-like behaviors by alleviating oxidative stress, neuroinflammation, and synaptic damage	Shen et al. (2021)
10. Gypenosides (>98% purity) *Gynostemma pentaphyllum*	Male C57/BL mice (8 weeks, 24 ± 2 g)	CUMS, 8 weeks	Oral gavage, once daily for 4 weeks	100 mg/kg	SPT, TST	*IL-1β, IL-6, TNF-α* (mRNA and protein), *p-NF-κB/NF-κB, p-IKKα/IKKα, p-IKKβ/IKKβ*, Iba1+ cell count (hippocampus)	Gynostemma pentaphyllum, clears heat and detoxifies, nourishes heart and calms spirit	Inhibits microglial activation and IKKα/IKKβ-NF-κB signaling in hippocampus	Gypenosides exerts antidepressant-like effects via suppression of hippocampal neuroinflammation	Dong et al. (2018)
11. Liquiritin*(Glycyrrhiza uralensis)*	Male ICR mice (8 weeks old, 20 ± 2 g)	Intraperitoneal injection of LPS (0.83 mg/kg)	Oral gavage	10 mg/kg and 20 mg/kg, once daily for 7 days	SPT, Forced swimming test	Hippocampal FGF-2 levels, Iba1+ cells, proinflammatory cytokines (*IL-1β, IL-6, TNF-α*), dendritic spine density	Licorice, relieves depression and cough, clears heat and detoxifies	Enhances FGF-2 to suppress neuroinflammation and maintain synaptogenesis	Liquiritin exerts antidepressant-like effects by enhancing FGF-2 to reduce neuroinflammation and protect synapses	Chen et al. (2020)
12. Curcumin (active component of *Curcuma longa*)	Mice, rats, pigs	Chronic stress, unpredictable mild stress, LPS, corticosterone	Oral, intraperitoneal, nasal (in novel formulations)	Dose-dependent effects observed; exact doses varied across studies	FST, TST, sucrose preference, social interactio*IL-6*n	*5-HT, DA, NE; BDNF; IL-1β, TNF-α, IL-6;* corticosterone; oxidative stress markers; *NLRP3*, Cyclooxygenase-2(COX-2), Inducible Nitric Oxide Synthase (iNOS)	Turmeric, relieves depression and cancer, clears heat and eliminates dampness	Modulates monoamines, reduces inflammation and oxidative stress, enhances neuroplasticity, regulates HPA axis and glutamate excitotoxicity	Curcumin exerts antidepressant-like effects through multiple pathways and shows promise as an adjunctive treatment	Ramaholimihaso et al. (2020)
13. Saffron essential oil (SEO) (*Crocus sativus L)*	Male ICR mice (25 ± 2 g)	CUMS for 28 days	nhalation via filter paper (4 h, twice daily)	2%, 4%, 6% (v/v, diluted with fractionated coconut oil)	OFT, SPT, TST, forced swimming test (FST)	Serum *5-HT, DA, GABA, BDNF;* hippocampal histopathology (*HE, Nissl); p-ERK, p-CREB1, BDNF,* etc. (WB)	Used historically in TCM for emotional disorders; aligns with “aromatic metabolites opening the orifices and calming the mind	Activation of the *MAPK-CREB1-BDNF* signaling pathway	SEO alleviates depression-like behaviors and hippocampal damage; 4% shows optimal efficacy	Chen et al. (2023)
14. Berberine*(Coptis chinensis Franch)*	Male Sprague-Dawley rats with diabetes and depression-like behavior	High-fat diet + Streptozotocin (STZ) injection + CUMS	Intragastric gavage	150 mg/kg/day (combined with ginsenoside Rb1)	FST,SPT, open-field test, EPM	Glucose metabolism, insulin resistance, plasma cortisol and ACTH, hippocampal *BDNF* expression, neuronal morphology (HE/Nissl staining)	Derived from Rhizoma Coptidis(Huanglian), traditionally used for diabetes with heat-clearing and damp-drying effects	Improves insulin resistance, reduces HPA axis hyperactivity, upregulates *BDNF*, protects hippocampal neurons	Berberine (with Rb1) improves depressive behaviors and glucose metabolism in diabetic rats	Zhang et al. (2021b)

#### 6.3.2 Baicalin (huang qin gan)

Baicalin, a flavonoid metabolite from *Scutellaria baicalensis Georgi* [Lamiaceae; Scutellariae radix], shows efficacy in behavioral tests (increased sucrose preference, reduced TST immobility). It promotes neuronal maturation and counteracts CUMS-induced deficits by activating the *Akt/FOXG1* signaling pathway (Zhang R. et al., 2019). Its neuroprotective effects also involve inhibiting the *GSK3β/NF-κB/NLRP3* inflammasome cascade (Zhang et al., 2018). In a CMS model, baicalin (50, 100 mg/kg, i.p.) alleviated depressive behaviors by modulating the Rac/LIMK/Cofilin pathway, which is crucial for cytoskeletal dynamics and neural resilience (Lu et al., 2019).

#### 6.3.3 Honokiol (from *Magnolia officinalis*)

Honokiol, a bioactive lignan isolated from *Magnolia officinalis*, demonstrated antidepressant-like effects in a corticosterone-induced rat model of depression by ameliorating HPA axis hyperactivity, enhancing hippocampal glucocorticoid receptor (GR) expression and GR/MR ratio, and attenuating systemic inflammation, as evidenced by reduced serum *IL-1β* levels (Zhang et al., 2020). In an LPS-induced depression model (1 mg/kg, i.p.), honokiol (10 mg/kg/day, p.o. for 11 days) attenuated neuroinflammation (reduced *TNF-α, IL-1β, IFN-γ*), inhibited IDO activity and quinolinic acid production, and prevented calcium overload in the brain (Zhang B. et al., 2019). Its clinical translation requires further systematic investigation.

#### 6.3.4 Icariin (from Epimediumspecies)

Icariin, a flavonoid metabolite from Epimedium species (e.g., Epimedium brevicornu Maxim. [Berberidaceae; Epimedii herba]), was studied in a perimenopausal depression model (partial ovariectomy plus CUMS). Icariin (12.5–50 mg/kg) ameliorated hormonal imbalances (increased E2, T; decreased FSH, LH), upregulated hypothalamic ERα, increased brain monoamines *(5-HT, DA, NE*), and activated ovarian *PI3K/AKT/Bcl-2* signaling, suggesting that modulation of this pathway may underlie its antidepressant-like effects in perimenopausal conditions (Cao et al., 2019).

#### 6.3.5 *Morinda officinalis* oligosaccharides (MOO)

*Morinda officinalis* oligosaccharides (MOO), derived from the root of *Morinda officinalis* How (Rubiaceae), have been investigated for their effects in a rat model of post-stroke depression (PSD). The PSD model was established by transient middle cerebral artery occlusion (tMCAO) followed by CUMS and social isolation. Oral administration of MOOs at 100 mg/kg/day for 2 weeks significantly ameliorated depressive-like behaviors, as evidenced by reduced immobility time and increased climbing time in the FST and TST. Mechanistically, MOOs suppressed *NLRP3* inflammasome activation in hippocampal microglia, leading to decreased levels of mature *IL-1β* and IL-18 and thereby attenuating neuroinflammatory responses. These findings highlight the potential of MOOs as a therapeutic candidate for post-stroke depression through modulation of microglia-mediated hippocampal inflammation. However, the impact of MOOs on cognitive impairments after stroke remains to be elucidated, as cognitive assessments were not included in this study (Li Z. et al., 2021).

### 6.4 Synthesis and visual summary

To concisely summarize the therapeutic effects and mechanisms discussed, the following sections will integrate the findings on TCM formulae, single botanical drugs, and purified metabolites. Graphical representations will be employed to illustrate the multi-target mechanisms and key pathways involved.

## 7 Systematic comparison and integration prospects of antidepressant mechanisms between traditional Chinese Medicine and western medicine

The accumulated evidence reviewed herein indicates that while both modern Western antidepressants and traditional Chinese medicine (TCM) aim to alleviate depressive symptoms, they are rooted in fundamentally distinct medical philosophies. These divergent paradigms manifest in their starting points, mechanistic logic, and target characteristics—yet they also present opportunities for synergy and complementary application in clinical practice (Table 3).

**TABLE 3:** Core mechanism comparison and clinical implications.

Feature	Western antidepressants (“sniper rifle” model)	Traditional Chinese medicine formulas (e.g., Chai-Shu-Jia-Yao, “shotgun” model)
Core hypothesis	Single-pathology model: e.g., monoamine deficiency (serotonin, norepinephrine)	Systemic imbalance model: dysregulation of multiple systems, including HPA axis, neuroinflammation, gut-brain axis, and emotional-physiological harmony (Zeng et al., 2025; Tang et al., 2025)
Drug type	Single chemical entity (e.g., fluoxetine, venlafaxine, ketamine)	Multi-metabolite herbal formula (e.g., Chai-Shu-Jia-Yao, Xiao-Yao-San) with dozens to hundreds of bioactive metabolites (Tang et al., 2025)
Target characteristics	High specificity: Clear and limited molecular targets (e.g., *SERT* inhibition by SSRIs, NMDA antagonism by ketamine) (Tang et al., 2025)	Low specificity, high complexity: Broad, network-wide modulation across multiple pathways and organ systems (“multi-metabolite, multi-target, multi-pathway”)
Intervention strategy	Precise and potent: Rapid suppression of core depressive symptoms. Designed to hit a defined biological target with high efficiency	Comprehensive and synergistic: Gradually restores systemic balance by simultaneously regulating mood, somatic symptoms (sleep, appetite, fatigue), and constitutional states (e.g., liver qi stagnation, spleen deficiency)
Onset of action	Relatively fast: Some agents (e.g., ketamine) exert antidepressant effects within hours to days (Tang et al., 2025)	Slower onset: Clinical improvement typically emerges over weeks, emphasizing sustained, holistic regulation (Xie et al., 2023)
Therapeutic effect	Strong efficacy for moderate-to-severe depression; highly effective in symptom suppression	Mild to moderate efficacy; excels in improving overall wellbeing, reducing relapse, and managing comorbid physical symptom
Side effect profile	Common and mechanism-linked side effects: e.g., sexual dysfunction (SSRIs), insomnia, weight gain, emotional blunting (Zhang et al., 2025)	Generally milder and less frequent side effects; better long-term tolerability. Adverse reactions are rare and often limited to mild gastrointestinal discomfort (Chen et al., 2016)
Individualization approach	Standardized dosing; limited personalization except in pharmacogenomic-guided prescribing	High individualization through syndrome differentiation (Zheng typing), tailoring formulas to the patient’s unique pattern (e.g., liver qi stagnation with spleen deficiency)
Philosophical basis	Biomedical reductionism: Focuses on correcting isolated biochemical deficits	Holistic systems theory: Emphasizes restoring dynamic equilibrium between mind and body, and between the individual and environment
Analogy	Sniper Rifle: A precise, powerful strike on a single target—effective when the aim is correct	Shotgun: Wide-ranging, simultaneous impact across a network—effective for diffuse, systemic dysfunction

The “sniper rifle” analogy illustrates the high specificity and rapid action of Western antidepressants on defined molecular targets (e.g., *SERT*, NMDA). In contrast, the “shotgun” metaphor represents the broad, synergistic, multi-metabolite, multi-target regulation of TCM, formulas across neural, endocrine, immune, and gastrointestinal systems.

### 7.1 Divergence in action paradigms: “precision intervention” under reductionism vs. “systemic regulation” under holism

Modern Western antidepressants are developed within a biomedical reductionist framework, characterized by “single-target, high-selectivity” pharmacological intervention. Their action typically originates at structurally defined molecular sites. For example, selective serotonin reuptake inhibitors (SSRIs) specifically bind to the *SERT*, leading to rapid elevation of synaptic 5-HT levels (Zhang et al., 2025).

In contrast, TCM treatment follows a holistic paradigm grounded in zheng differentiation (syndrome pattern identification), aiming for systemic rebalancing through multi-metabolite, multi-target interactions. A single TCM formula often contains dozens to hundreds of bioactive plant metabolites, which collectively modulate multiple pathological axes—including the monoaminergic system, HPA axis, neuroinflammation, oxidative stress, neurotrophic signaling, and gut microbiota (Zeng et al., 2025). Loganin demonstrates significant antidepressant-like effects in a CUMS-induced murine model by normalizing monoamine neurotransmitter levels, ameliorating HPA axis hyperactivity, and upregulating hippocampal BDNF expression (Guo et al., 2023). Chai Shao Jie Yu Granules exert therapeutic benefits through synergistic “multi-metabolite, multi-target, multi-pathway” actions, resulting in comprehensive regulation of both neuroendocrine and neurotransmitter systems (Tang et al., 2025).

Danzhi Xiaoyao San (DXS) and its active metabolites exert antidepressant effects through multi-target and multi-pathway mechanisms, including the modulation of monoamine neurotransmitters, suppression of neuroinflammation, and restoration of neuroplasticity (Matsuzaka and Yashiro, 2025), Similarly, various TCM botanical drugs and their active metabolites, such as seahorse (Lievanos-Ruiz and Fenton-Navarro, 2025), and Vanillin, as an aromatic substance with a pleasant odor, has been shown to exert antidepressant effects that are closely associated with the regulation of monoamine neurotransmitter levels in the brain (Ma et al., 2025), Acupuncture exerts its antidepressant effects primarily through multiple pathways, including regulating neurotransmitter balance, enhancing synaptic plasticity, inhibiting neuroinflammation, modulating the neuroendocrine system (such as the HPA axis), and improving gut microbiota and tryptophan metabolism (Pardossi et al., 2024). The water extract of Gastrodia elata and its main constituents, gastrodin and 4-hydroxybenzyl alcohol, exert antidepressant effects by modulating the monoaminergic neurotransmitter system and promoting neuronal cytoskeletal remodeling (Chen et al., 2016).

## 8 Scientific basis and future directions for integrated therapy

The purported mechanistic differences between traditional Chinese medicine (TCM) and Western pharmacotherapies in treating depression are often framed as complementary, summarized under the principles of “efficacy enhancement and side effect reduction.” However, this harmonious narrative risks obscuring the persistent lack of robust, causal evidence underpinning the biological plausibility of many TCM interventions. Rather than assuming synergy as inherent, we must subject it to rigorous empirical testing: do TCM agents genuinely modulate defined neurobiological pathways, or are observed benefits attributable to non-specific effects, expectancy bias, or methodological limitations in current preclinical and clinical studies?

Conventional antidepressants—while effective for many—act primarily through well-characterized mechanisms involving monoaminergic transmission and HPA axis regulation (Chen et al., 2016; Krystal et al., 2023).

In summary, mounting evidence supports the view that depression is not a single, uniform condition but rather a heterogeneous syndrome shaped by complex interactions among neuroinflammation, bioenergetic stress, synaptic dysfunction, and dysregulated glia–neuron communication. Within this network, purinergic signaling—driven by ATP and adenosine—has emerged as a key integrative hub. Dysfunctional P2X7 receptor activation, impaired astrocytic ATP release, and aberrant A2A receptor activity have all been mechanistically linked to depressive phenotypes across diverse experimental models, highlighting their potential as novel therapeutic targets (Ren et al., 2025; Ribeiro et al., 2019).

Saikosaponins (SSs) from Bupleurum scorzonerifolium Willd., administered orally at doses of 120–400 mg/kg, significantly ameliorate behavioral deficits in LPS-induced murine models of depression. The core mechanism involves the direct binding to and downregulation of the P2X7 receptor, thereby inhibiting *NLRP3*/Caspase-1/GSDMD-mediated microglial pyroptosis. This paradigm exemplifies a novel approach in modern pharmacology for the multi-metabolite, multi-target intervention of psychiatric disorders using traditional herbal medicines (Bi et al., 2025). Salidroside, a bioactive metabolite isolated from Rhodiola rosea, exhibits robust antidepressant-like effects at doses of 20–50 mg/kg in rodent models, particularly under chronic stress conditions. Nevertheless, many existing studies on herbal extracts fail to define the precise bioactive metabolites, confirm direct target engagement, or genetically validate mechanistic pathways—limitations that highlight the importance of rigorous pharmacological validation, as partially addressed in this study (Chai et al., 2022). Nevertheless, many studies fail to identify the exact bioactive fractions, determine target engagement, or validate mechanisms using genetic or chemogenetic tools.

The real challenge—and opportunity—lies in integrating these observations into a unified framework grounded in systems biology. Could specific TCM agents intersect with the purinergic axis? Might saikosaponin D, salidroside, or CHSGS modulate *P2X7/NLRP3*-driven neuroinflammation, restore astrocyte-dependent ATP dynamics, or rebalance adenosine-mediated inhibition (Ren et al., 2025)? These hypotheses require testing not through behavioral correlation alone, but via target-specific validation using:

Cell-type-specific knockout models (e.g., P2X7R inmicroglia) to determine necessity of key signaling nodes; Chemogenetic or optogenetic approaches to assess circuit-level engagement; Multi-omics profiling (transcriptomics, metabolomics, microbiome) to map system-wide impacts (Pardossi et al., 2024; Xu et al., 2015).

AI-driven network pharmacology to predict botanical drug -metabolite-target interactions and inform rational combination strategies. Even non-pharmacological modalities—such as acupuncture and herbal baths—are clinically promising for symptom relief (Liu et al., 2023; Gan and Liu, 2024; Haiyan, 2017) demand deeper mechanistic scrutiny. Are neurochemical changes reproducible? What are the pharmacokinetic profiles of active agents?

Ultimately, the integration of TCM and Western medicine must move beyond philosophical alignment or clinical observation alone. It must be anchored in transparent, falsifiable, and reproducible science—demanding improved standardization of herbal preparations, rigorous experimental design, and shared data platforms. By grounding combined therapy in mechanism-driven understanding—informed by modern discoveries such as purinergic dysregulation—we stand not merely to manage symptoms, but to build a future of evidence-based, personalized, and globally scalable treatment for depression.
